# Mechanical Effect of an Implant Under Denture Base in Implant-Supported Distal Free-End Removable Partial Dentures

**DOI:** 10.3390/dj12110358

**Published:** 2024-11-11

**Authors:** Naomichi Murashima, Yoshiyuki Takayama, Toshifumi Nogawa, Atsuro Yokoyama, Kiwamu Sakaguchi

**Affiliations:** 1 Hokkaido University Hospital, Kita14, Nishi5, Kita-ku, Sapporo 060-8648, Japan; 2 Department of Oral Functional Prosthodontics, Division of Oral Functional Science, Graduate School of Dental Medicine, Hokkaido University, Kita13, Nishi7, Kita-ku, Sapporo 060-8586, Japan; takayama@den.hokudai.ac.jp (Y.T.); t.nogawa@den.hokudai.ac.jp (T.N.); yokoyama@den.hokudai.ac.jp (A.Y.); sakaguti@den.hokudai.ac.jp (K.S.)

**Keywords:** implant-assisted removable partial denture, removable partial denture, finite element analysis

## Abstract

**Background**: In recent years, implant-assisted removable partial dentures (IARPDs) have been used clinically. However, the extent to which additional implants reduce the burden of supporting tissues is unclear. The aim of this study was therefore to investigate the influence of implanted IARPDs on stress sharing among supporting tissues, using finite element (FE) analysis. **Methods**: FE models were constructed based on the computed tomography (CT) of a patient with a unilateral defect of the mandibular premolars and molars and the surface data of an RPD with cuspids as abutments, designed using computer-aided design software. A titanium implant was placed in the area equivalent to the first premolar, second premolar, or first molar (IARPD4, IARPD5, and IARPD6, respectively). FE analysis was performed for laterally symmetrical models, i.e., bilateral distal free-end IARPDs. A vertical load of 200 N was applied to the central fossa of the artificial premolars or molars (L4, L5, or L6). **Results**: Equivalent stress in the alveolar mucosa and vertical displacement of the denture was smaller, with IARPDs under L5 and L6 loads, compared to RPDs. However, abutment teeth were displaced upward under an L6 load in the IARPD5 model. **Conclusions**: Within the limitations of this study, the area corresponding to the first molar was recommended as the location for an implant under the denture base of bilateral distal free-end IARPDs. Implants located in the area corresponding to the second premolar may apply non-physiological extrusion force on abutment teeth under the load on the artificial second molar.

## 1. Introduction

The hypothesis of this study is that in mandibular IARPDs, the greatest reduction in supportive tissue burden is in IARPDs with implants in the first molar when loaded in the premolar region. Removable partial dentures (RPDs) are generally used to restore oral function in partially edentulous patients. However, RPDs can occasionally cause a wide variety of problems, such as the insufficient recovery of the function, the overload of the abutment teeth, and the resorption of the residual ridge [1,2].

Dental implants (hereafter abbreviated to “implants”) are widely used and recommended as one of the best alternatives to RPDs for partial edentulism due to their good masticatory function and patient satisfaction [3]. However, implants place demands on the amount and quality of bone and involve surgical invasiveness [4].

On the other hand, implant-assisted RPDs (IARPDs) have been applied clinically and have been reported to be a better prosthodontic treatment, with higher patient satisfaction [5,6,7]. Further, the applicability of shorter implants can decrease surgical invasiveness and expand indications [8,9]. The implant survival rate in IARPDs is reported to be 93.1%, and clinical outcomes are considered favorable [10].

Model studies have reported that an implant on the distal side of the denture base can reduce its distal displacement [11]. It has also been reported that unilateral free-end ISRPDs, placing the implant farther from the abutment tooth, have an advantage with respect to bending stresses on the implant and the displacement of the abutment tooth [12]. Finite element analysis (FEA) demonstrated that IARPDs with implants placed in the molar region caused favorable stress distribution in the peri-implant bone, framework, and implant area [13,14]. Stress in the framework at the bilateral free-end IARPDs has also been reported [15].

Matsudate et al. reported that the implant farther from the abutment tooth made the vertical stress on the abutment tooth greater, and one closer to the abutment caused greater lateral force on the abutment of the unilateral free-end IARPDs [16].

However, it has also been reported that implants closer to the abutment teeth have a more appropriate stress distribution in the supporting tissues [17] and that the use of a distal guide plane reduces stresses on the bone and the abutment teeth [18]. Ohyama et al. reported that the higher the healing abutment height, the smaller the displacement of the denture [19].

From the above, the mechanical advantages of IARPDs remain unclear, and there is no clear clinical evidence. Furthermore, many of the mechanical studies to date have not been based on anatomical models, and it is not clear whether they have been accurately reproduced clinically. The aim of this study was therefore to investigate the influence of implants under the saddle of distal extended RPDs on stress sharing among abutment teeth and the residual ridge using finite element (FE) models based on computed tomography (CT) images.

The clinical significance of this study is in its determination of the mechanical factors associated with the long-term prognosis of supportive tissues by examining the effective location of an implant under occlusal load to reduce the burden of supportive tissues.

## 2. Materials and Methods

### 2.1. FE Models

**Figure 1:**
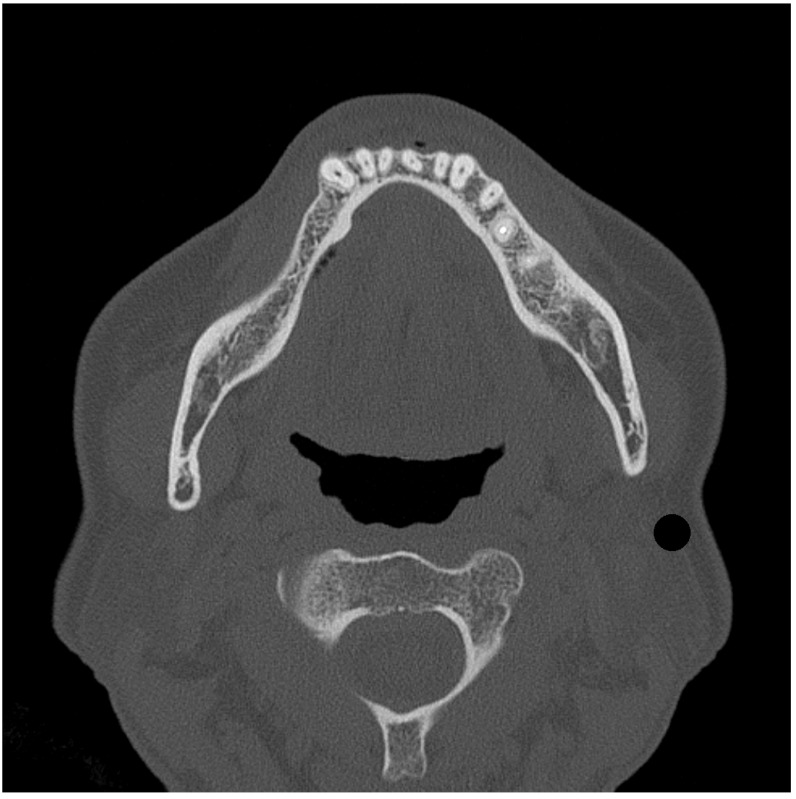
CT data (DICOM) for mandibular bone.

This study used CT images of a patient (age 53) with a right unilateral defect of the mandibular premolars and molars who had visited the Department of Removable Prosthodontics at Hokkaido University Hospital (Figure 1). The bone and tooth surfaces of the right half were extracted from CT images encoded in the Digital Imaging and Communications in Medicine (DICOM) format according to their CT values via medical image processor computer-aided design (CAD) software (Materialise Mimics Medical 21.0; Materialise, Leuven, Belgium).

**Figure 2:**
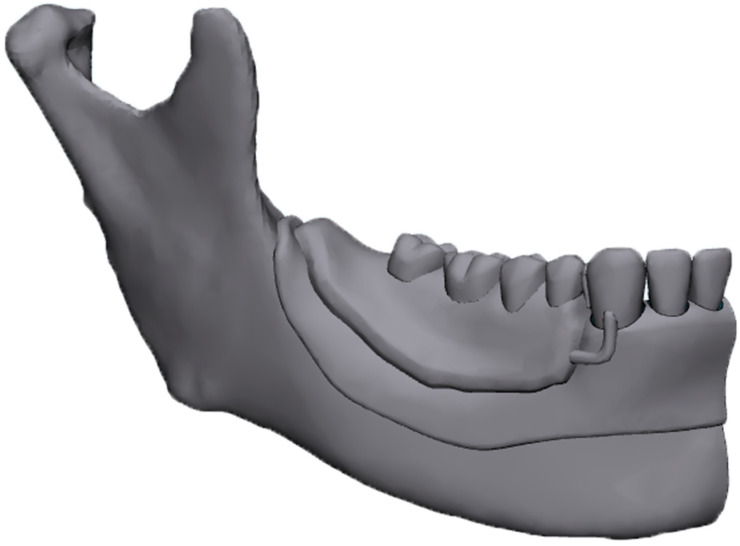
CAD model (STL format).

**Figure 3:**
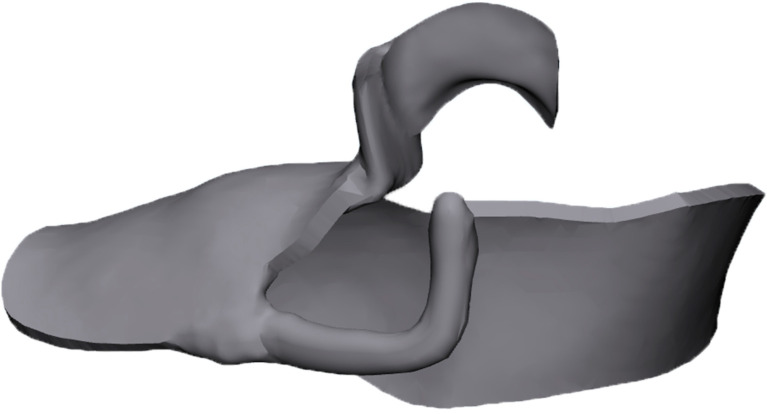
The denture metal frame was designed with a 3D CAD solution for RPD frameworks (DIGISTELL; C4W/DIGILEA, Montpellier, France) and imported with CAD software (Materialise 3-matic Medical 12.0; Materialise, Leuven, Belgium). The Co-Cr framework consisted of a set comprising a cingulum rest, an I-bar clasp, and distal proximal plate on each abutment tooth.

The outline of the periodontal ligament (PDL) and mucosa was constructed with CAD software (Materialise 3-matic Medical 12.0; Materialise) from the surfaces of teeth and alveolar residual bone, respectively [20]. Metal frameworks of RPD and IARPDs were designed using three-dimensional (3D) CAD solutions for RPD frameworks (DIGISTELL; C4W/DIGILEA, Montpellier, France) (Figure 2). The Co-Cr framework consisted of set comprising a cingulum rest, an I-bar clasp, and a distal proximal plate on each abutment tooth (Figure 3) [14,15,17].

A cylindrical dental implant made of titanium, 3.5 mm in diameter and 8.5 mm in width, was placed under the denture saddle in the region of the first premolar, second premolar, or first molar in the model (IARPD4, IARPD5, and IARPD6, respectively). In addition, a model without an implant (RPD) was constructed as a control. These CAD data were outputted as standard triangulated language (STL) files.

**Figure 4:**
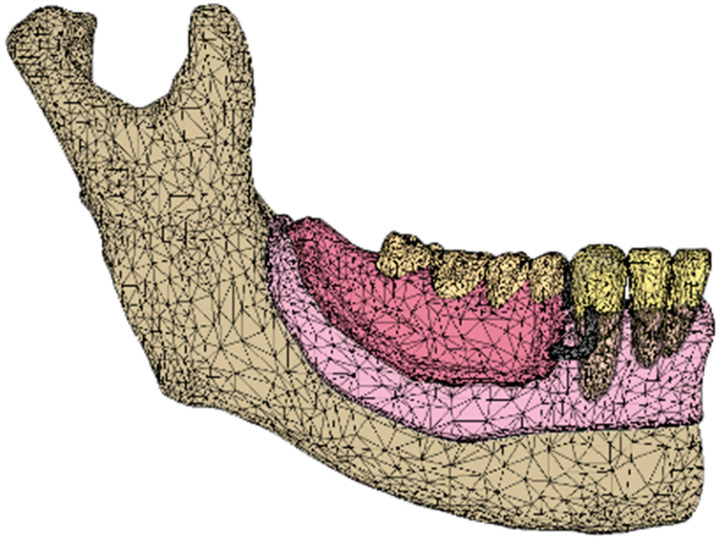
Bilateral distal free-end RPD FE model (nodes: 11,203; elements: 48,586). Material properties are displayed in Table 1 [21,22,23,24]. All materials were assumed to be isotropic and elastic.

All the above-mentioned surfaces were imported into FE analysis (FEA) pre–post processor software (Marc. Mentat2010; MSC Software, Newport Beach, CA, USA) and converted into FE models (Figure 4). FEA was performed as a bilateral distal free-end RPD (Figure 1) by constraining the lateral movement of the nodes in the mid-sagittal plane.

The material properties are indicated in Table 1.

### 2.2. Condition of Contact

Conditions of contact at the mucosa–denture base, denture base–implant, and retainer–abutment tooth interfaces were considered in the analysis as “gap” elements, which connected two nodes of each surface. This element resists compression but not tension. Friction at these interfaces, except for the retainer–tooth interface, was neglected, considering the lubricant effect of saliva. The coefficient of friction at the retainer–tooth interface was determined to be μ = 0.05 [25,26,27] and was incorporated into the applicable gap element.

### 2.3. Boundary Conditions

**Figure 5:**
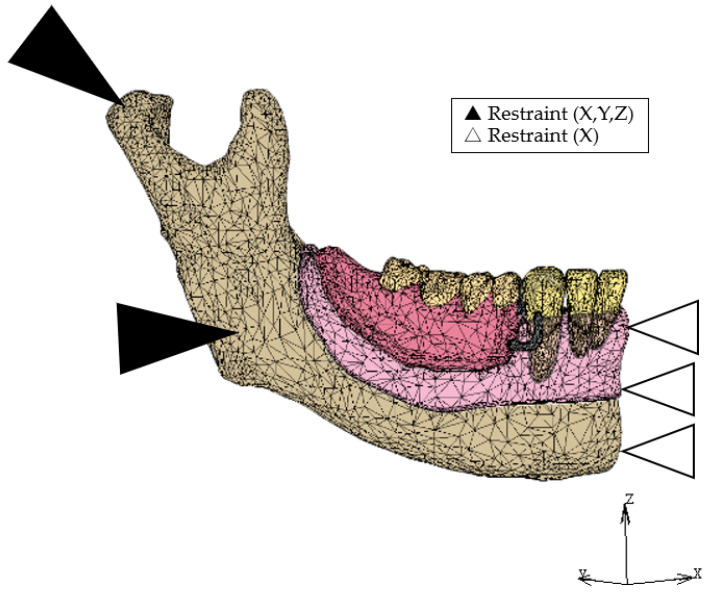
Boundary condition.

**Figure 6:**
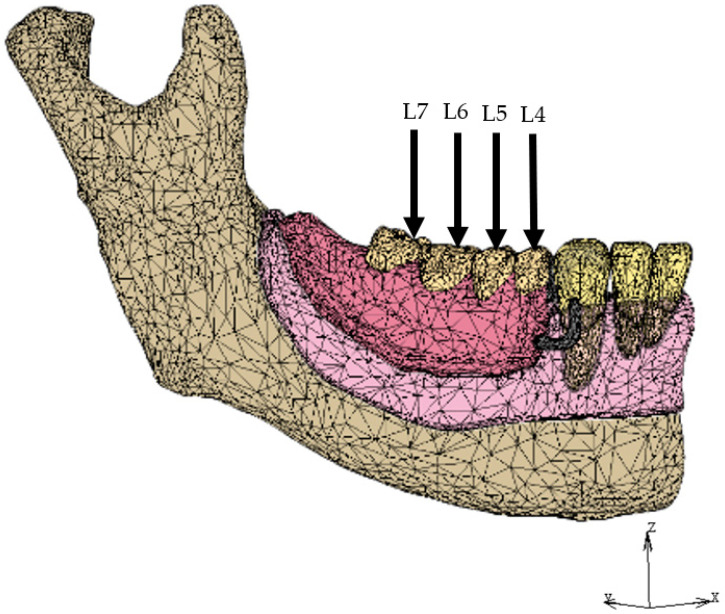
Load point.

Displacement was restricted at each node on the top of the condyle and the stop of the masseter muscle in all directions, and at all nodes in the cross-section of the midline of the mandible in the lateral direction (Figure 5). A vertical load of 200 N was applied to one of the loading points located on the point corresponding to the first premolar (L4), second premolar (L5), first molar (L6), and second molar (L7) (Figure 6) [14,28]. In the IARPD models (IARPD4, IARPD5, and IARPD6), we selected the amount of initial space between the top of the implant and the denture base so that they are in contact with each other under 100 N of vertical load on L6 (Table 2) [28,29].

### 2.4. Evaluation of Analysis

We evaluated each model in terms of the relative vertical displacement of the denture base at the distal end and the apex of the abutment tooth (canine) with reference to the nearest node on the bone surface. We also evaluated the distribution of von Mises-equivalent stress in the mucosa of the alveolar ridge.

## 3. Results

### 3.1. Equivalent Stress in Alveolar Mucosa (Figure 7, Figure 8, Figure 9 and Figure 10)

In all models, stress was largest under the L7 load, compared with other load conditions. IARPD6 showed the greatest effectiveness in reducing stress by placing an implant compared with an RPD.

Likewise, displacement of the denture base was the largest under L7 loading (Figure 11). As the position of an implant became more distal, displacement decreased proportionally.

**Figure 7:**
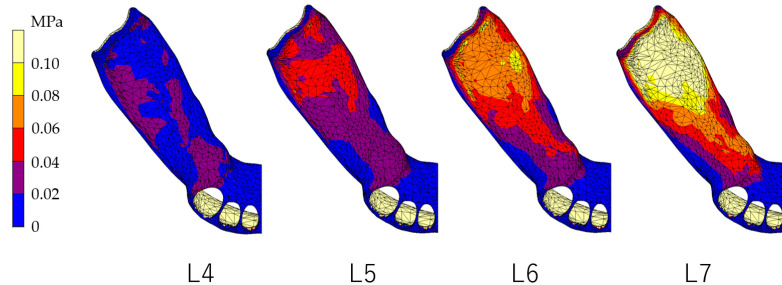
Equivalent stress in the mucosa area (RPD).

**Figure 8:**
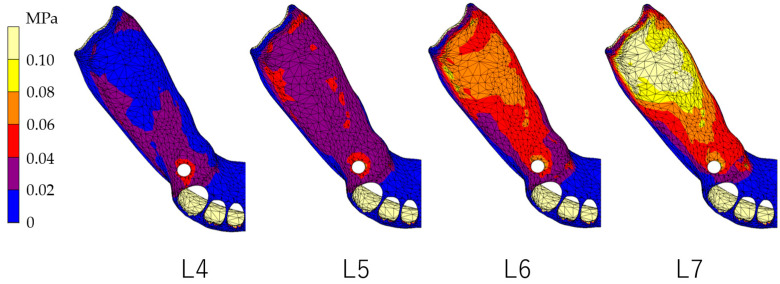
Equivalent stress in the mucosa area (IARPD4).

**Figure 9:**
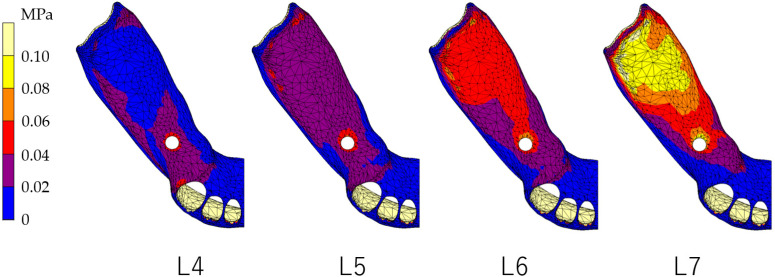
Equivalent stress in the mucosa area (IARPD5).

**Figure 10:**
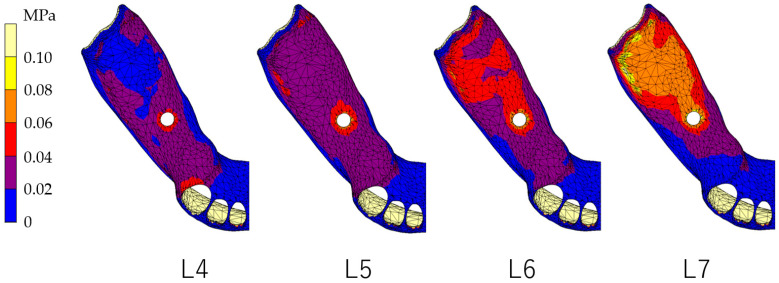
Equivalent stress in the mucosa area (IARPD6).

**Figure 11:**
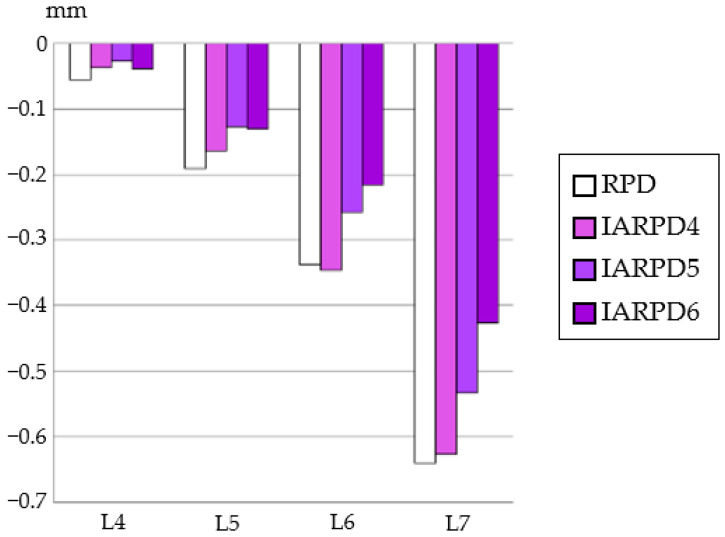
Displacement of denture.

### 3.2. Equivalent Stress in PDL (Figure 12, Figure 13, Figure 14 and Figure 15)

The RPD model showed decreased stress in the PDL under the L7 load compared to the L4, L5, and L6 loads. However, the IARPD models showed the greatest decrease under L6 loading. Compared with the RPD model, most IARPD models showed reduced stress in the PDL, except for IARPD5 under an L7 load.

Figure 16 shows vertical displacement at the apex of the canine. In the RPD model, vertical displacement of the canine became smaller as the loading point was located more distally. On the other hand, in all IARPD models, the canine displaced upward under L7 loading.

**Figure 12:**
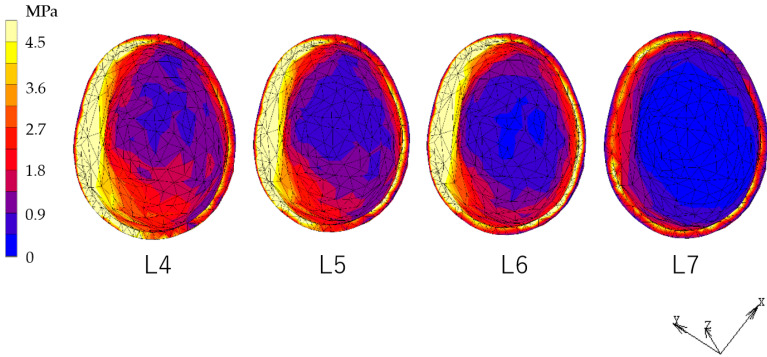
Equivalent stress in the canine PDL (RPD).

**Figure 13:**
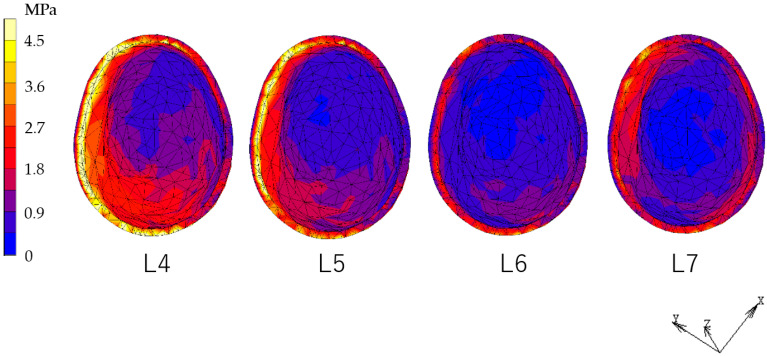
Equivalent stress in the canine PDL (IARPD4).

**Figure 14:**
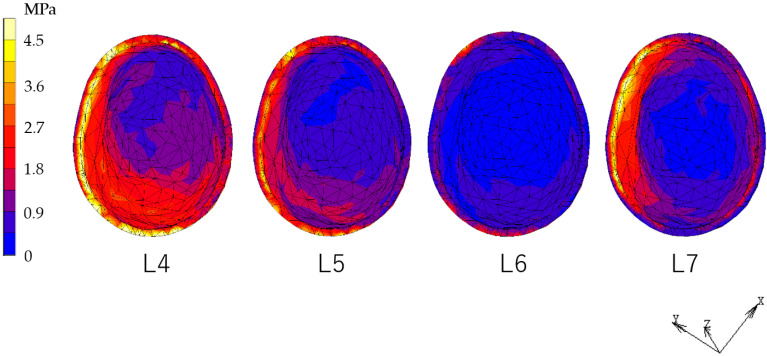
Equivalent stress in the canine PDL (IARPD5).

**Figure 15:**
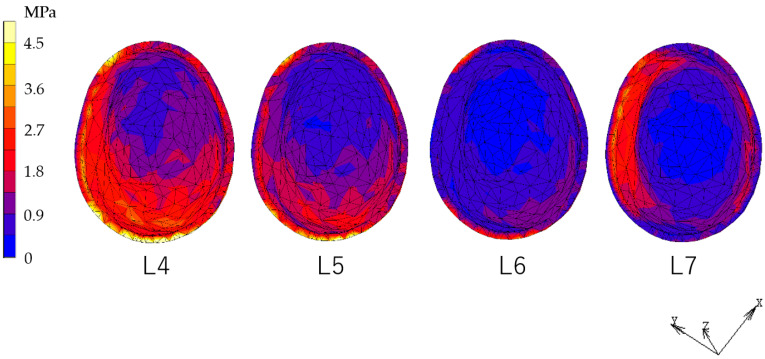
Equivalent stress in the canine PDL (IARPD6).

**Figure 16:**
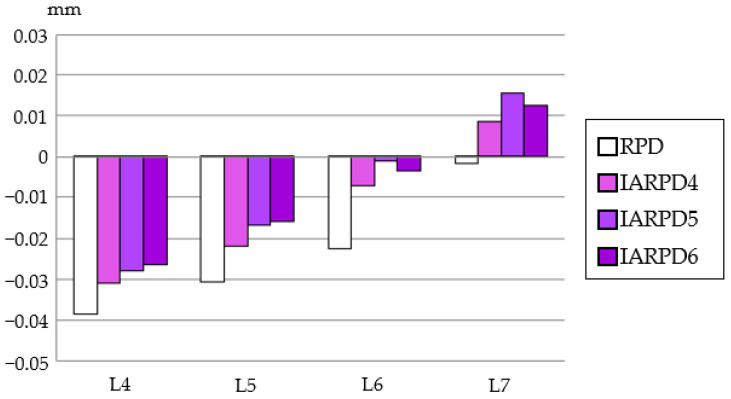
Displacement of the apical area of the canine.

## 4. Discussion

The findings of this study showed that when implants were placed in the second bicuspid region, overloading in the centric position resulted in an upward load on the abutment tooth. This may deform the clasp arm and reduce its retention.

Masticatory efficiency in patients wearing a free-end RPD is not sufficiently recovered because the denture base sinks far more than the abutment teeth and stress on the abutment tooth increases accordingly [1,23,30,31]. Clinical application of the IARPDs has been proposed to suppress displacement of the denture base in such cases [5,6].

Several reports have examined the biomechanical behaviors of abutment teeth and implants of IARPDs under occlusal loading [11,16]. Moreover, FE models generated from CT images have recently become common. However, only a few studies have conducted stress analyses with precise FE models of IARPDs based on CT images because of the complexity of shapes, material properties, and boundary conditions, including contact analysis [15,20]. Since this study used CT images from only one patient, results may depend on the case. Morphological aspects such as the status of the abutment teeth, root length, and residual ridge, the biomechanical properties of residual tissues, and surgical techniques such as impression methods and denture adjustment could affect the results.

With respect to the implant location, a more distal location was favorable because of the effective reduction in stress on the mucosa and the PDL of the abutment teeth. However, a more distally located load than the implant lifted the abutment teeth. This displacement was due to the lever effect, with the implant acting as a fulcrum. However, in the IARPD4 model, this effect was negligible because of the smallest movement of the denture base around the implant, which was near the rest of the retainer in the canine.

Matsudate et al. described abutment tooth being lifted in a model study of unilateral free-end IARPDs with the implant located medially to the second premolar tooth [16].

The present results also corresponded with a report by Ohyama et al. [19]. They stated that the implant under the denture base reduced the displacement of the abutment teeth under a mesially located load because displacement of the denture base was suppressed in unilateral free-end IARPDs.

On the other hand, given the results from this study, locating an implant in the second molar area could be preferable because displacement of the denture base and abutment teeth was speculated to be more effectively suppressed than IARPD6. However, in this case, insufficient bone height from the mandibular canal prevented implant placement at that site. In such a case, IARPDs with mini-implants has been reported to reduce the burden on the supporting tissues [32]. Although a shorter implant would be applicable, the effects on IARPDs should be investigated more in the future.

Our FE model had rough elements for the implant and bone to maintain analysis time within the limit of the hardware use because the purpose of this study was to investigate the burden on the abutment tooth and mucosa. Thus, the occlusal pressure burden on the implant and surrounding bone could not be evaluated in this study. To evaluate the distribution of stress in implants and surrounding bones under IARPDs, models need to be constructed with the precise shape of the implant and surrounding bone, including the thread.

The amount of initial space was determined so that the top of the implant would be in contact with denture base under an occlusal force during mastication of approximately 100 N. Since displacement of the mucosa is much larger than that of the implant, the behavior of the denture base under occlusal forces smaller than 100 N was basically the same as that of conventional RPDs [28,29]. Therefore, if the relief was small, the implant would make contact under smaller occlusal force and support most of the occlusal force. Conversely, too much relief would result in the behavior of the denture base being the same as that of conventional RPDs unless stronger occlusal force is applied. The result may therefore depend on the amount of space. This study was performed under a vertical load. However, functional occlusal loading has lateral components because masticatory movements contain lateral components [33]. We are therefore planning to investigate the mechanical effects of inclined loading on the crestal mucosa and abutment teeth.

It has been reported that the use of an ERA-type attachment (Sterngold Dental, Attleboro, MA, USA) reduces the burden on the supporting tissues [16] and that patient satisfaction is higher with magnetic attachments than with healing abutments [34]. On the other hand, Putra-Wigianto et al. reported no significant impact on implant survival or patient satisfaction with the use of attachments, and the effect of attachments on IARPDs is still unclear [35]. The effect of attachments on IARPDs should be investigated in the future.

## 5. Conclusions

Within the limitations of this study, the area corresponding to the first molar was recommended as the location of an implant under the denture base of bilateral distal free-end IARPDs. An implant located in the area corresponding to the second premolar can cause non-physiological extrusion force on the abutment teeth via the load on the artificial second molar.

## Figures and Tables

**Table 1:** Material properties [21,22,23,24].

		Elastic Modulus (MPa)	Poisson’s Ratio
Bone (coronal bone)		13,700	0.30
Mucosa		0.5	0.45
Tooth	Enamel	80,000	0.30
	Dentin	17,600	0.25
Co-Cr alloy		211,000	0.3
Denture (acrylic resin)		2200	0.31
Titanium		110,000	0.33
PDL		2.3	0.49

**Table 2:** Amount of initial space between implant and denture.

	IARPD4	IARPD5	IARPD6
Initial space (mm)	0.06122	0.09335	0.12698

## Data Availability

The data supporting the conclusion of this article will be made available by the authors on request.
